# Applying a WASH Risk Assessment Tool in a Rural South African Setting to Identify Risks and Opportunities for Climate Resilient Communities

**DOI:** 10.3390/ijerph19052664

**Published:** 2022-02-25

**Authors:** Thandi Kapwata, Zamantimande Kunene, Bianca Wernecke, Samantha Lange, Guy Howard, Anisha Nijhawan, Caradee Y. Wright

**Affiliations:** 1 Environment and Health Research Unit, South African Medical Research Council, Johannesburg 2094, South Africa; Thandi.Kapwata@mrc.ac.za (T.K.); Zama.Kunene@mrc.ac.za (Z.K.); Bianca.Wernecke@mrc.ac.za (B.W.); 2 Department of Environmental Health, Faculty of Health Sciences, University of Johannesburg, Johannesburg 2006, South Africa; 3 Water and Health Research Centre, Faculty of Health Sciences, University of Johannesburg, Johannesburg 2006, South Africa; Samantha.Lange@ekurhuleni.gov.za; 4 Department of Civil Engineering, Cabot Institute, University of Bristol, Bristol BS8 1TU, UK; Guy.Howard@bristol.ac.uk; 5 Department of Civil Engineering, University of Bristol, Bristol BS8 1TU, UK; Anisha.Nijhawan@bristol.ac.uk; 6 Environment and Health Research Unit, South African Medical Research Council, Pretoria 0001, South Africa; 7 Department of Geography, Geoinformatics and Meteorology, University of Pretoria, Pretoria 0001, South Africa

**Keywords:** environmental health, climate change, pathogens, sanitation services, sustainable development, water supply

## Abstract

Climate change threatens the health and well-being of populations. We conducted a risk assessment of two climate-related variables (i.e., temperature and rainfall) and associated water, sanitation and hygiene (WASH)-related exposures and vulnerabilities for people living in Mopani District, Limpopo province, South Africa. Primary and secondary data were applied in a qualitative and quantitative assessment to generate classifications of risk (i.e., low, medium, or high) for components of hazard/threat, human exposure, and human vulnerability. Climate-related threats were likely to impact human health due to the relatively high risk of waterborne diseases and WASH-associated pathogens. Vulnerabilities that increased the susceptibility of the population to these adverse outcomes included environmental, human, physical infrastructure, and political and institutional elements. People of low socio-economic status were found to be least likely to cope with changes in these hazards. By identifying and assessing the risk to sanitation services and water supply, evidence exists to inform actions of government and WASH sector partners. This evidence should also be used to guide disaster risk reduction, and climate change and human health adaptation planning.

## 1. Introduction

Water, sanitation, and hygiene (WASH) is seen as the foremost preventative programme against transmission of waterborne and water-washed diseases, including, for example, cholera, and giardia, which cause diarrhea and nausea [1]. Combining provision of safe sanitation with potable water supplies for consumption and personal hygiene can reduce the prevalence of these diseases. These actions can assist in reducing diarrhoeal diseases, especially among vulnerable groups such as children under five years of age [2].

Diarrhea is a symptom of infections caused by a host of bacterial, viral, and parasitic organisms, most of which may be spread by water contaminated with faeces or by transmission via food, hands, and flies because of poor hygiene and sanitation [3]. Infection is more common when there is a shortage of safe water for drinking, cooking, and cleaning, and inadequate sanitation and hygiene. Water contaminated with human faeces from sewage, septic tanks and latrines is of particular concern. Animal faeces, which may find its way into human water supplies, also contain microorganisms that can cause diarrhea, for example the excreta-related pathogen *Cryptosporidium* which has been identified as one of the most common causes of diarrhea in young children globally [3]. Additionally, unsafe domestic water handling and storage are also important risk factors. In 2016, the absence of adequate WASH programs and practices was estimated to lead to 829,000 global deaths from diarrhea (60% of the total diarrhea deaths) including 297,000 deaths in children under five years of age, equivalent to 5.3% of all deaths in this age group [4].

In South Africa, diarrhea is one of the top two leading causes of death (the other being respiratory diseases) in children under five years of age [5] where poor sanitation, lack of personal hygiene and inadequate water supplies [6] are contributing factors. In rural areas, pit latrines remain the primary toilet facilities and in-yard or communal standpipes are the primary modes of water provision [7]. Low levels of service, combined with unreliability in water supply and poor maintenance of latrines, increases human exposure to pathogens that cause waterborne and water-washed diseases [4].

Over and above these environmental health risks, climate change, including global warming and changes in extreme weather events, may threaten the health and well-being of rural communities. South Africa is expected to see an increase in average daily ambient temperatures by ~4 to 6 °C by 2100 (at a low mitigation scenario, or A2) [8]. Rural areas in northern parts of South Africa may experience extremes of rainfall leading to periods of flooding as well as dry spells and/or drought [9]. Such climatic circumstances have been shown to impact the occurrence of water-borne diseases [10]. 

Monitoring climate factors that affect the WASH sector, as well as WASH-associated pathogens, is essential for the protection of public health [11]. One of the ways in which this can be done is to undertake risk assessments. These help determine hazards or threats (defined as a dangerous phenomenon, substance, human activity or condition that may cause loss of life, injury or health impacts, property damage, loss of livelihoods and services, social and economic disruption and environmental damage) to which communities are exposed, community vulnerabilities (defined as the characteristics and circumstances of a community that make is susceptible to the damaging effects of a hazard), and potential exposure, defined as the proportion of the community that could be adversely affected by the hazards. A risk assessment uncovers opportunities and address weaknesses to improve preparedness and response.

In a previous study called iDEWS (infectious Disease Early Warning System project), hospital admissions data for diarrheal disease were analyzed using contour analysis along with temperature and rainfall data to look for coincidences between health outcome and extremes in temperature and/or rainfall. Children under five years of age were especially vulnerable to diarrhea when summertime conditions were wetter than usual [12]. Ikeda et al. [12] found an anomalously higher number of diarrhea cases during ‘warmer than usual’ conditions in the dry winter season. An anomalously higher number of diarrhea cases were found during ‘drier than usual’ conditions for the winter (the dry season in Limpopo) and spring (the season when rain may begin in Limpopo). 

**Figure 1:**
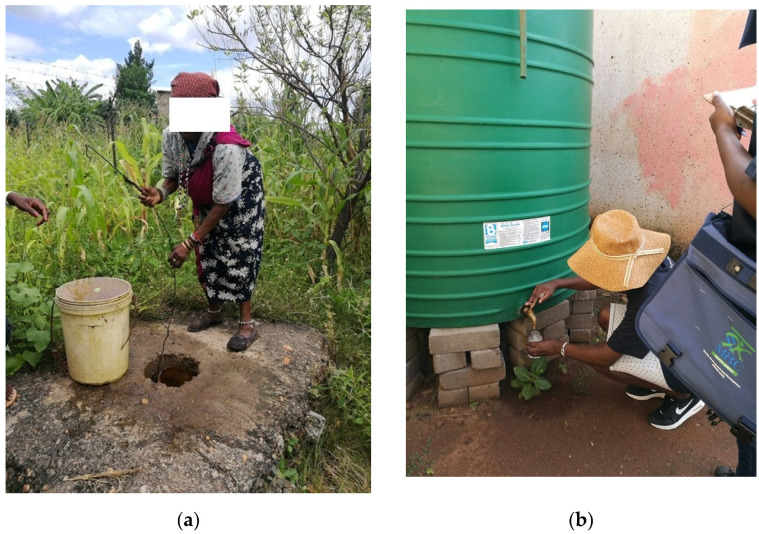
(**a**) A borehole and (**b**) stored water in a personal water tank (unit) called a ‘JoJo’ in a village in Giyani, Mopani District (Photos are author’s own).

iDEWS also interviewed households from four villages in Mopani District and a sub-sample of households provided water samples for analyses from their drinking water source—including boreholes (Figure 1a), indoor taps, standpipes, and water stored in containers and storage units (Figure 1b) [6].

One in five households reported that there had been at least one case of diarrhea in their household during the previous summer. Most used sources of drinking water were a standpipe (inside the yard) (45%) followed by an indoor tap (at least one) inside the dwelling (29%). After adjusting for confounders, the occurrence of diarrhea was statistically significantly associated with using water from an indoor tap (Adjusted Odds Ratio (AOR): 2.7, 95% CI: 2.1–6.5) [6]. *Escherichia coli* was most often detected in water samples from standpipes and kitchen water storage containers. 

Dry conditions during winter were associated with diarrhea in children under five years of age [12] and may lead to increased water storage, in non-optimal containers, raising the risks of water contamination. Reduced use of water for personal hygiene and cleaning of outdoor pit latrines also affected sanitation quality. A shortfall of the iDEWS project was that, while potable water quality was assessed, it was not possible to determine the pathogens likely to be contributing to the occurrence of diarrhoeal disease. Here, we attempt to fill this gap by considering hospital and clinic collected specimen data for the Mopani District. The aim of our research was to investigate whether Mopani District is facing climate threats that may compromise WASH projects and programs and, compounded by additional vulnerabilities, whether this puts the communities living in the district at risk of exposure to WASH-related diseases. We achieved this aim via two study objectives: (1) we explored laboratory specimen data to understand the range of bacterial waterborne pathogens likely to be associated with adverse health outcomes among Mopani communities; and (2) we conducted a risk assessment of climate-related threats and associated WASH-related personal exposure and vulnerabilities to provide evidence to support decision-making for resilient communities in the rural areas of Mopani District.

## 2. Materials and Methods

### 2.1. Study Area

**Figure 2:**
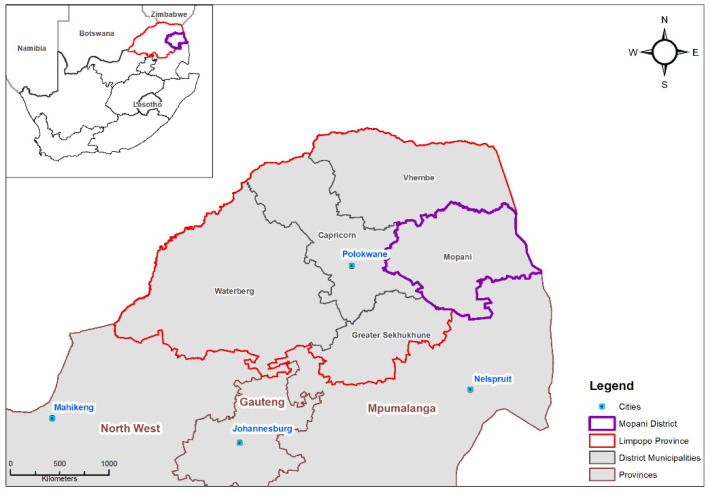
Location of Mopani District (outlined in purple) in Limpopo province, South Africa.

The Mopani District Municipality is in north-west Limpopo and is home to about 20% of the province’s population (Figure 2). Maximum and minimum temperatures in this area range from 29 °C and 24 °C, and 24 °C and 11 °C in summer and winter, respectively [12]. Mean precipitation is highest during the summer months of December, January, and February (19.5 mm) and lowest during winter (June, July, and August, 0.6 mm) [12].

In 2019, there were about 600,000 people living in poverty across the District (based on the upper poverty line) and 16% of the population was unemployed [13]. Only a third of the population had access to piped water inside their yards, about 16% had access to a flush toilet or a chemical toilet, and 76% of the population used pit latrine toilets [13].

### 2.2. Study Design

The United Nations Children’s Fund (UNICEF)/Global Water Partnership (GWP) WASH Climate Resilient Development Risk Assessments for the WASH conceptual framework was applied [14]. This framework was deemed as the most suitable given its applicability for climate resilience risk assessments. A combination of the UNICEF/GWP Part 1 (high-level assessment for risks across all types of hazards)/Part 2 (a detailed assessment for climate-specific risks only) scope and method of risk assessment was considered. While other risk assessment approaches exist, this international method was deemed reputable, reproducible, and appropriate with the four criteria of hazard, exposure, vulnerability, and capacity. We conducted this high-level assessment for two climate variables (deemed the hazard or threat), namely high temperatures/heatwaves, rainfall/flooding and dry spells for the Mopani District.

### 2.3. Patient Data

Results of anonymized patient samples tested for bacterial pathogens from 2015 to 2018 and sent from 83 hospitals and clinics in Mopani District to the National Health Laboratory Service (NHLS) for analysis were acquired with permission. Specimen samples testing positive for one or more bacterial pathogens (only bacterial pathogens were available) and associated with waterborne and water-washed diseases were considered. Pathogens were included for reasons specified in column 4 of Table 1 and based on their relationship with WASH. The indicators applied in the exposure scoring exercise identified people that could be adversely affected by the hazards using published data and the NHLS dataset bacterial pathogens to which the laboratory sample population had been exposed. Using an arbitrary classification, exposure was deemed high if >5% of the laboratory sample population were affected, medium if 0.5–5.0% of the laboratory sample population were affected and low if <0.5% of the laboratory sample population were affected.

### 2.4. Risk Assessment Approach

Rainfall as a climate hazard for Mopani District was classified using two characteristics—present-day frequency and intensity. This was done using data from published studies. A similar approach was taken to score the frequency and intensity of high temperature and drier than usual/dry spells.

For the high-level assessment, we used available literature from previous studies carried out in the Mopani District that described historical meteorological data and, where possible, future projections for temperature and rainfall. Then, we analyzed the specimen laboratory data to consider the types of bacterial pathogens in all urine and stool samples processed over a period of four years from hospitals and clinics in Mopani District. This provided an indication of the range of bacterial pathogens to which people living in Mopani District were exposed. We also extracted data for WASH-related variables from the last South African National Census [16] which were used to assess exposure and vulnerability. These variables included water supply and sanitation, and socio-economic factors, respectively.

The final risk classification of each hazard/threat, exposure and vulnerability component was evaluated to understand the impacts of three meteorological variables: high temperature/heatwaves; heavy rains/flooding; and drier than usual/dry spells on potential exposure and vulnerability of people living in Mopani District. Research ethics clearance for access to data was granted by the South African Medical Research Council Research Ethics Committee (EC005-3/2014).

The goal of the assessment was to identify risks to human health associated with exposure to WASH-related pathogens and to consider these risks in a climate change context. The risk assessment method applied in this study helps to broaden the understanding of risk to encompass all the different hazards/threats that could affect the WASH sector. Risk arises because of the interaction of hazard/threat, exposure, and vulnerability [14].

**Figure 3:**
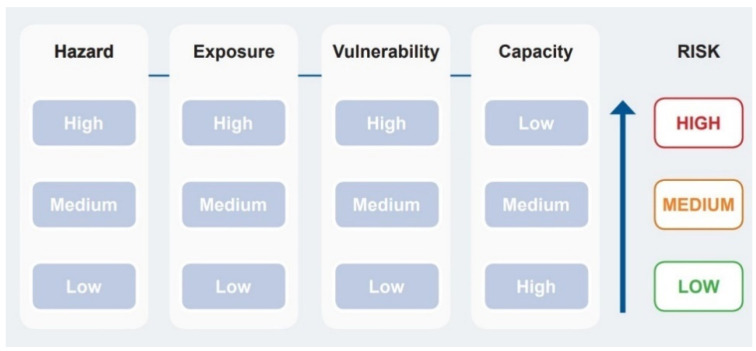
The impact of hazard, exposure, vulnerability, and capacity (not performed here) on the overall risk score (adapted from [14] page 5).

The risk assessment considers the nature and extent of risk from hazards/threats and evaluates existing conditions of vulnerability that may potentially harm exposed people in terms of property, services, livelihoods, and the environment on which they depend for their livelihoods [14]. We considered primary and secondary data in a qualitative and quantitative assessment to generate individual classifications of risk for hazards/threats, human exposure, and vulnerability. Like previous climate change and WASH assessments [11,14] each component for hazard/threat, exposure and vulnerability was assigned a classification for risk using a three-point Likert scale (i.e., low, medium, high) depending on the data and evidence. The scores were assigned by applying the framework for risk as outlined in Figure 3 of UNICEF/GWP [14].

Risk increases as vulnerability increases, for example, in the instance of an extreme weather event such as flooding of a rural village. While Figure 3 includes capacity in the overall risk score, in our assessment it was not possible to include capacity. Capacity usually exists at a regional or local government level; little information is available on community capacity or how well people prepare, respond, recover, and learn in relation to risk. We adopted the high-level assessment approach for discussing capacity elements arising from the risk assessment results and used these to discuss implications for the communities, for example, social communication tools, civil society responsibility, adequate budget allocations, partnerships etc.

#### 2.4.1. Hazard Scoring

Three hazards/threats were assessed: heatwaves/extreme heat, flooding/heavy rainfall, and dry spells. Hazards/threats were scored according to scales described above depending on their main characteristics. These included present-day and expected future frequency, intensity, geographical extent, and season, depending on available literature and data. Decisions on the classifications were made with expert moderation among the project team.

#### 2.4.2. Exposure Scoring

The exposed population included the people living in the Mopani District of Limpopo province likely to be exposed to heatwaves/extreme heat, flooding/heavy rainfall, and dry spells. Data were collected from published studies and national health surveys.

#### 2.4.3. Vulnerability Scoring 

The following components were considered: Physical, e.g., resilience of WASH infrastructure; Environmental, e.g., waste disposal; Human, e.g., population age, employment status, level of income; and Political and Institutional, e.g., WASH and related policies, government effectiveness. Data for the physical, environmental, and human components of vulnerability were obtained from the 2011 South African Census [17]. These included data on service delivery (waste disposal services), socio-demographic characteristics (age and socio-economic conditions) and sanitation (types of latrines).

Information on the political and institutional component was gathered by searching for government and/or municipal documents on climate change policies and adaptation plans for water supply and sanitation sectors of Mopani District [18]. This component was written up and scored/classified using guidance developed by the University of Bristol on scoring policy indicators through a review of sector policies, climate change policies and adaptation plans [11]. The components were assigned a classification of low, medium, or high depending on how they influenced the susceptibility of the population to the hazards/threats. 

#### 2.4.4. Risk Summary

Risk was determined from the findings for hazards/threats, exposure, and vulnerability. Since we only had three main hazards, heatwaves/extreme heat, flooding/heavy rainfall, and dry spells, we did not rank final risk classifications; instead, we discuss how the findings help understand challenges and opportunities that may improve resilience in the WASH sector in Mopani District.

## 3. Results and Discussion

### 3.1. Hazards/Threats Classification

**Heatwaves/extreme heat**: Results are presented in Table 2. In Mopani District, mean maximum and minimum temperatures from 2002 to 2016 differed by season, with the highest maximum being in summer and the lowest minimum in winter, as expected. The range in outdoor temperature was large throughout the seasons; for spring, summer, and winter, mean outdoor temperature ranges were 18–40 °C, 21–41 °C and 19–33 °C, respectively [6].

Engelbrecht at al. [8] used Representative Concentration Pathway 8.5 [19] climate modelled data for 2080–2099 and projected increases in monthly temperature to assess possible future estimates of outdoor temperature in the Mopani District. Average ambient temperature was estimated to increase by a mean of 4–6 °C and higher temperatures may occur in what were previously considered cooler seasons of spring and winter [8].

**Flooding/heavy rainfall and dry spells**: The Mopani District lies in a summer rainfall area and mean weekly precipitation ranges from <1 mm per week during winter to 19 mm per week during summer [12]. Despite the area generally being dry all year round, it does experience periods of flooding. In some instances, the flooding is due to tropical cyclones that cause landfall across Mozambique and move north-west into Limpopo Province, e.g., the flood disaster of 1999/2000 was a result of Cyclone Eline [20]. The heavy rainfall experienced in Mopani District could increase the leaching of harmful substances into the environment and contribute to the development of stagnant pools of flood water that promotes the growth and development of pathogens which increases potential human exposure and adverse waterborne infections. 

Linkages between the frequency and intensity of droughts and flooding have been seen in the El Nino Southern Oscillation (ENSO) phenomenon [21]. The flood disaster that occurred in December 2010–January 2011 was linked to a La Nina event and caused massive displacement of people and numerous deaths. Crops, infrastructure, and property were also affected/damaged.

A study that used two CORDEX-Africa regional climate models (i.e., CanESM2m and IPSL-CM5A-MR) considered projected changes in precipitation in the Limpopo River basin [22]. It found that the basin is likely to experience reduced streamflow in the near and distant future, as well as frequent dry and wet conditions that can be interpreted as drought and flash floods, respectively.

### 3.2. Exposure Scoring

Mopani District Municipality is home to ~1,160,000 people who are potentially exposed to the three climate hazards/threats described above. There are about 340,000 households; 14% have flush toilets connected to a sewerage system; and 13% have piped water into their dwelling [35].

**Findings of the urine and stools samples:** Potential exposure of the Mopani District communities to bacterial pathogens was estimated using laboratory data. Of the total number of specimens (*n* = 20,250, for 2015 to 2018) received from the NHLS, 3070 specimens were considered WASH-related pathogens (Table 3). *Escherichia Coli* was the most identified organism. Unfortunately, the NHLS did not have date per specimen taken, so a time series of the data was not possible. 

About 70% of samples stemmed from patients between the ages of 19 and 65 years of age and more than twice as many patient samples were from women, compared to men. There was a 15% prevalence of WASH-related bacterial pathogens in the laboratory data. According to the classification of exposure, this is considered high since >5% of the laboratory sample population were affected (based on our arbitrary classification) (Table 4).

Data from the South African 2015/2016 District Health Barometer [35] showed that the burden of diarrhoeal disease is high in Mopani. The District reported a case fatality rate (i.e., child deaths in health facilities) due to diarrhea of 4%. This is above the national target of 3% and was classified as high exposure.

An assessment of drinking water quality of 192 water samples collected from four villages in Mopani District found that 27% of water samples from standpipes [6] did not meet international WHO guidelines [36] or South African Water Quality Guidelines [37] for domestic water due to the concentrations of *Escherichia coli*. Therefore, almost a third of homes were at risk of waterborne diseases and the exposure was classified as high.

### 3.3. Vulnerability Scoring

Most of the population of Mopani District have their own means of disposing of waste, do not have access to piped water, and is reliant on pit latrines (Table 5). Therefore, this population is classified as highly vulnerable in relation to the waste disposal and technology of the WASH physical infrastructure components of vulnerability scoring. The pit latrines are not often well constructed and are not designed to be resilient to seasonal variation or seasonal shocks, such as high rainfall events. Flooding of these latrines increases possible exposure to disease-causing pathogens leading to a rise in illnesses [38,39]. Individuals using such facilities are at a disproportionately high risk of sanitation-related diseases including diarrhea, gastroenteritis, and hookworm, compared to those who use piped sewerage facilities [40]. Therefore, the population of Mopani District is vulnerable to these diseases due to the prevalence of poorly maintained pit latrines. Furthermore, unemployment is high in Mopani District and poor communities are less likely to be able to afford to implement climate-proofing strategies such as building raised latrines to protect against heavy rains and floods [41]. The use of pit latrines is also a source of water pollution—in Limpopo groundwater in most boreholes was contaminated by human waste from pit latrines [42].

**Figure 4:**
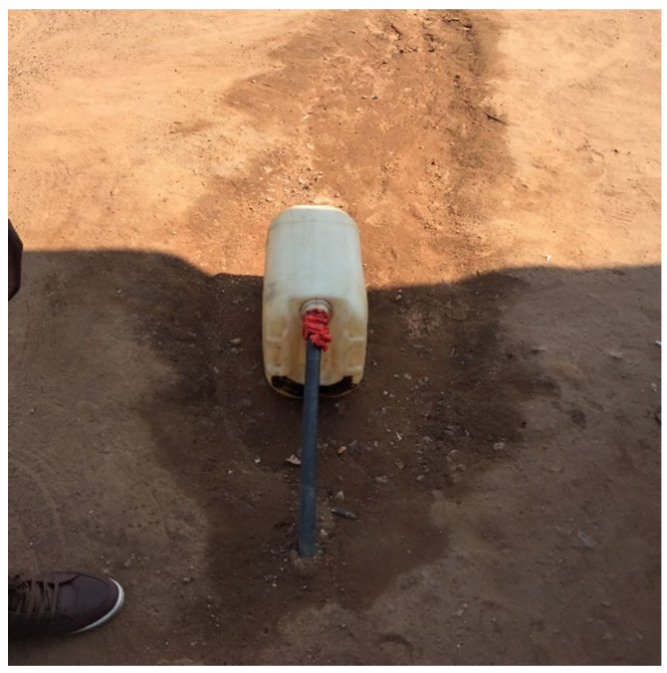
A pipe coming out from the ground that sporadically has water running out from it in Mopani.

About 16% of the population had no access to formal, piped municipal water. For example, water comes out of a pipe that emerges from the ground in between dwellings (Figure 4). This might increase the chances of exposure to contaminated water sources which in turn may increase the risk of disease. However, even when households had access to water from an indoor tap, the water was contaminated as shown in previous research in Mopani. Water from water treatment plants may not comply with standards, or pipes may have leaks leading to contaminated drinking water piped into dwellings.

Parts of the Mopani population may be less likely to practice regular handwashing, given the challenges of water access, which has been reported to significantly reduce disease transmission and risk [33]. Furthermore, keeping hydrated is one of the most important ways to prevent heat-related illness [44] therefore the lack of access to water places the residents of Mopani at risk of the negative health impacts during hot days or heatwaves.

Population growth is another factor that is likely to put pressure on water supplies. Table 5 shows that almost 50% of the population of Mopani District is under 50 years of age. Therefore, in the future, more children will reach childbearing age, increasing the demand for safe, reliable water supplies. Other factors placing pressure on water supplies are heavy rainfall and increased flooding that could cause damage to water utility infrastructure [10] thus placing more people at risk of disrupted water supplies.

Our review of climate change policies and adaptation plans for the water supply and sanitation sectors for Mopani District (Table 5) found that policies discuss the risks of climate change on these sectors, but they do not provide recommendations or a budget for adaptation. The Service Delivery Budget and Implementation Plan (SDBIP) for 2019–2020 for Mopani District [45] defines monthly capital expenditure for water and wastewater in the Mopani District but this is not in the context of climate change—if such a change in location of WASH project budgets were made, a sense of urgency to attend to WASH projects may emerge. Therefore, the district was scored as being highly vulnerable to political and institutional factors that influence climate change policies and adaptation plans for the water supply and sanitation sectors.

Our research suggests that high temperatures/heat waves and flooding have frequently occurred in the Mopani District in the past, and these could have had possible effects on the health of communities due to the high risk of waterborne diseases shown in the exposure risk assessment. We further identified several vulnerabilities that increase the susceptibility of the population to these adverse outcomes including environmental, human, physical infrastructure, and political and institutional elements. The classification of vulnerabilities showed that it is people of low socio-economic status in Mopani who are least likely to cope with changes in high temperatures, heavy rainfall and drier than usual/dry spells that may be linked with climate change. Therefore, reducing inequalities and alleviating poverty could reduce the vulnerability of communities in the Mopani District to WASH and climate change-related adverse health impacts.

The population of Mopani is also vulnerable to the impacts of climate-related threats due to the lack of climate change policies and adaptation plans for the water supply and sanitation sectors. These policies lack detail and do not provide sufficient information beyond the proposal for additional research or early research stages. The climate change policy discusses how climate change is likely to impact water availability and drinking quality. Although policies acknowledge the importance of adaptation measures for the water supply in Mopani, no recommendations are provided and although the source of funding for adaptation measures is identified, no budget is specified. Identification of climate change-related risks, planning of interventions, allocation of budget and execution of plans on the ground are essential steps to climate-proof or build resilience of the population, as people are already being affected by climate impacts, and will be more so in the future. 

In Mopani, there are several hygiene and sanitation concerns that also affect WASH health outcomes—climate change is one additional stressor. A key concern is personal hygiene, such as regular cleaning of water storage containers and pit latrines. This is evidenced by a self-reported questionnaire that showed only 7% of respondents washed their households and cleaned the toilet daily, and 67% cleaned it weekly [6].

Overall, our study found that Mopani District is facing climate threats that may compromise WASH projects and programs and compounded, by its vulnerability, this puts it at risk of exposure to WASH-related diseases.

### 3.4. Study limitations

We purposefully selected the UNICEF/GWP risk assessment tool. Other tools do exist and may find different results. It was assumed that the NHLS data was of good quality, but no verification of findings was possible. Our focus was on water-borne (bacterial) pathogens; however, since we considered urine and stool samples, we cannot isolate the possibility that some samples may have contained food-borne pathogens since some of the pathogens are carried via both pathways. It was difficult to reconcile data for synthesis and comparison given the different data sets cited in the literature and the different spatial scales at which data were available. An attempt was made to be as consistent and thorough as possible in the reporting of the literature.

Our assessment was largely based on published studies for Limpopo province, or the Mopani District and this information provided some insight into the lack of quantitative research related to extreme heat and hydrological events (i.e., floods and dry spells). For example, there were very few studies that investigated hydrological hazards or their associated trends. There was also a lack of published information on the severity, frequency and intensity of heatwaves or episodes of extreme heat in Mopani. The same was true when searching for geographically relevant material on drier than usual/dry spells for the area.

## 4. Managerial Insights

WASH is a collective term that refers to the provision of safe drinking water, access to adequate sanitation, good hygiene practices, and improved water resource management. Addressing the interdependent shortcomings that affect each of these various factors could contribute significantly to the reduction of WASH-related diseases. To facilitate this, information on WASH indicators such as those described in our paper should be collected regularly at various administrative levels. This can be done through inspections of sanitary services, analysis of microbial water quality and community surveys. Such routine monitoring is crucial for identifying challenges as they occur so that interventions can be developed in a timely fashion. Supply should also be monitored and evaluated against measures that enhance a reliable supply of safe water and prioritize the protection of human health. The analysis and interpretation of such data will inform technical interventions, legislative and regulatory processes and provide the basis for targeted hygiene education.

Our study provides an understanding of the state of WASH services that contributes to the lack of progress in the district of Mopani towards meeting the requirements for Sustainable Development Goal 6, “Ensure access to water and sanitation for all” [46]. In addition, our results could also be used to assess the level of preparedness of Mopani to manage disaster risk due to natural and man-made hazards according to the Sendai Framework for Disaster Risk Reduction, by providing a description of the environmental and health risks that human-induced climate change variables such as extreme heat and rainfall pose in the district [47].

## 5. Conclusions

Mopani District needs community-wide sanitation improvement programs that are tailored to local conditions and the social context. Effective water quality management as a government-managed service needs urgent attention, both in terms of supply and quality. Piped water is not sufficient; the water must meet local and international quality standards. Environmental Health Practitioners working in these communities should be trained to initiate awareness programs to assist households to reduce exposure and transmission of pathogens causing ill health. WASH projects and programs should be well budgeted for, preferably aligned with climate change activities, to increase the sense of urgency needed to protect communities, especially during extreme weather events.

## Figures and Tables

**Table 1:** Description of each bacterial pathogen, its likely disease symptoms/disease outcomes, and likely linkage to WASH. All pathogens were included here based on their inclusion in the World Health Organization Microbial Fact Sheets [15], except for *Alcaligenes Faecalis* which is not on the fact sheet but was deemed relevant for inclusion in this study.

Bacterial Pathogen	Source of Exposure	Symptoms/Diseases	WASH Description
*Burkholderia* *Cepacia*	Hygiene	Lung infections	The germs spread either by direct contact, such as kissing, or indirectly from touching objects on which germs were situated, such as doorknobs. This is known as cross-infection and can happen in social settings such as events, gatherings, or meetings.
*Enterococcus* *Faecalis*	Hygiene	UTIs, prostatitis, intra-abdominal infection, cellulitis, wound infection	A strong association between hand hygiene method and bacterial contamination of hands has been found
*Enterococcus* *Faecium*	Hygiene	UTIs	A strong association between hand hygiene method and bacterial contamination of hands has been found
*Enteropathogenic Escherichia Coli (EPEC)*	Water, food, hygiene	diarrhea	In general, strategies for the prevention and control of the spread of EPEC should include access to safe water, good handling practices to reduce the risk of food contamination, sanitation measures, public education, and vaccination
*Non-specific pathogenic Escherichia Coli*	Water, food, hygiene	Diarrhea, UTIs, respiratory illness, pneumonia	Un-speciated *E. coli* may not be pathogenic but, given that the laboratory records state its presence, it may be taken to represent a pathogenic strain.
*Salmonella Group*	Water, food, and hygiene	Gastrointestinal illness	Some common ways in which a person can become infected with salmonella include: Eating food or drinking water that has been contaminated with animal faeces Eating food that has been handled by a food worker who has not properly washed their hands
*Shigella Flexneri*	Water, hygiene	Diarrhea	Swallowing recreational water (for example, lake or river water) while swimming or drinking water that is contaminated with stool (faeces) containing the germ.

**Table 2:** Classification of rainfall and temperature as climate threats in Mopani.

Hazard/Threat	Present-Day Frequency and Intensity	Classification of Hazards
High temperature/heatwaves [21,22,23,24,25,26]	Mopani District experiences warm temperatures all year round. Temperatures increased between 1958–2017, mostly remaining above the long-term mean 23. Most reports about high temperatures and heatwave events in South Africa are from media releases and news media: Heatwave reported in Limpopo in December 2018 affecting mainly Vhembe and Mopani Districts. Temperatures exceeded 40 °C. In 2015, Mopani District faced a water crisis with up to 90,000 residents experiencing water shortages due to a week-long heatwave that dried up the main dam. In 2013, severe heatwaves resulted in a devastating drought that affected the Mopani District. Heatwave days during the period 1971–2000 showed heat wave duration ranged from 2–10 days with the highest number of days being observed in Mopani and Vhembe Districts.	High: The Mopani District is well known for experiencing heatwaves and high temperature and adequate data exist to support this classification.
Heavy rains/flooding [27,28,29,30,31]	Flood warnings developed by Mozambique classify streamflow as an extreme flood event when flow anomalies exceed 1.5 standard deviations above the short-term mean. Extreme precipitation/streamflow events for Limpopo basin using this definition showed that residents can expect an average of one or two extreme precipitation events each year and that flood warning levels exceeded 25–27 days each year between 1998–2003. Mopani experienced wet seasons frequently between 1961 and 2011—mean seasonal rainfall (Oct to Mar) was 122.6 mm and rainfall above 100 mm was recorded 75% of the time. Seasonal rainfall anomalies found that Mopani experienced particularly wet seasons frequently (every ~1–2 years) from 1971–2011. Mopani received the highest amount of rainfall compared to all other districts in Limpopo throughout the year from 1998–2017. Most rain was received during summer months (Dec, Jan, Feb). Mean interannual variability of precipitation showed that frequency of extreme flood events had increased between 1958–2017. In Jan 2012, a state of disaster was declared in Mopani District following severe flooding. More than 500 mm of rainfall was recorded in 24 h.	High: The Mopani District is well known for experiencing heavy rains and flooding and adequate data exist to support this classification.
Drier than usual/dry spells [12,32,33,34]	Mopani has a dry winter season between the end of May and the beginning of September. Dry years (i.e., below average rainfall) tend to cluster together over extended periods in the Limpopo River Basin. Nearly half of the time there is some form of drought (or drier than usual conditions) in Mopani, mostly linked to the El Nino phenomenon. An anomalously high number of diarrhea cases during ‘drier than usual’ conditions for winter (dry season in Limpopo) and spring (season when rains begin in Limpopo) occurred. In July 2019 news reports claimed dams were left empty, taps ran dry after political elections, and boreholes stopped working.	Medium: Existence of dry spells is evident but additional data for the Mopani District is needed to assign that this as high since drought also probably requires detailed consideration.

**Table 3:** Descriptive statistics of laboratory specimens with presence of bacterial pathogens for Mopani District.

Variable	Frequency n	Frequency %
**Number of laboratory samples by** **WASH-related organisms (n = 3 070)**		
Alcalgenes Faecalis Subsp faecalis	12	0.4
Burkholderia Cepacia	19	0.6
Enterococcus Faecalis	642	21
Enterococcus Faecium	108	6
Enteropathogenic Escherichia Coli	1	<1
Escherichia Coli	2241	73
Salmonella Group	34	1
Shigella Flexneri	9	0.3
Shigella Group	4	0.1
**Patient age**		
Under 5-year-olds	308	10
5–18-year-olds	335	11
19–65-year-olds	2019	66
Older than 65 years	263	9
Missing	143	5
**Patient gender**		
Male	923	30
Female	2124	69
Missing	23	<1

**Table 4:** Scoring of exposure for Mopani District.

Human Indicator	Outcome for Mopani District	Classification of Exposure
**Child health**	Mopani District had the second highest number of deaths due to diarrhea nationally for children aged under 5 years of age (case fatality rate of 4%) [35].	High: Above the national target of 3%.
**Morbidity**	In 2015, 15% of hospital admissions from two major hospitals in the district were diarrhea related [12].	High: More than 15% of total admissions were due to a WASH-related illness.
	From 2015 to 2018, among 20,250 laboratory specimens, there was a 5% prevalence of WASH-related bacterial pathogens (this study).	High: 5% of laboratory specimens were associated with a WASH-related bacterial pathogen.
**Water quality**	Microbial water quality of water samples collected from Mopani standpipes had high microbial risk. Total coliform counts exceeded 100 counts/100 mL water in 29% of these samples. A similar pattern was observed for *E.coli* with more than 20 counts/100 mL detected in 11% of samples from standpipes [2,3,6].	High: More than a third of households were exposed to microorganisms that can cause diseases and that may come via piped water provided by the municipality.

**Table 5:** Scoring of vulnerability for the Mopani District. Note. ^#^ In 2020, the average monthly wage in the formal non-agricultural sector was ZAR23 133 [43].

**Factor**	**Element**	**Question**	**Outcome for Mopani District**	**Classification of Vulnerability**
Environ-mental	Waste disposal	Is domestic waste collected and disposed of safely by municipal authorities?	Only 15% of population has refuse removed by authorities. 68% of population use their own refuse dump.	High: A large proportion of the population does not have access to formal domestic waste disposal.
Human	Age of population	Is there a large population of very old or young people?	40% of the population is under the age of 18 years.	Medium: Close to 50% of the population is under the age of 18 years.
	Socio-economic stability	What are the levels of employment? What are levels of income per month? ^#^	26% of population is employed; 50% are not economically active. 17% are unemployed. 43% of population do not earn any income; 45% earn less than ZAR 1600; 4% earn between ZAR 1600–3200; 2% earn between ZAR 3200–6400; 4% earn more than ZAR 6400.	High: There is low socio-economic stability in Mopani District. High percentages of the population do not earn any income or earn well below minimum wages.
Physical	Technology of WASH physical infrastructure	Which latrine types are predominantly used? Are they resilient?	69% of population in Mopani use pit latrines. These are often poorly designed and not designed to be resilient to climate shocks.	High: Poor/basic WASH infrastructure is available. Resilience of infrastructure is low due to poor design and construction of pit latrines.
		What is the availability of water supply infrastructure?	16% of population is without access to piped water.	High: The percentage of people without access to piped water from a tap in their home, yard or community is high (>80%).
Political and institutional	Climate change policies and adaptation plans for the water supply and sanitation sectors for Mopani district	Are there any government or municipal policies/legislature on climate change and adaptation plans for water supply and sanitation sectors?	Climate change policies and adaptation plans identify the risks to sanitation and drinking water, but sanitation policies do not discuss climate change or offer recommendations on adaptation.	High: The Mopani District Municipality Climate Change Vulnerability Assessment and Response Plan identifies sanitation as an impacted sector. Projections suggest increased risk of flooding and rainfall intensities, flash floods and regional flooding, litter and debris blocking water and sanitation systems. No sanitation policy was found.

## Data Availability

The laboratory sample data are available from the National Health Laboratory Services. All hazard-related data were extracted from publicly available manuscripts as cited in the text.
